# An improved random forest-based computational model for predicting novel miRNA-disease associations

**DOI:** 10.1186/s12859-019-3290-7

**Published:** 2019-12-03

**Authors:** Dengju Yao, Xiaojuan Zhan, Chee-Keong Kwoh

**Affiliations:** 1 School of Software and Microelectronics, Harbin University of Science and Technology, Harbin, 150080 China; 2 College of Computer Science and Technology, Heilongjiang Institute of Technology, Harbin, 150050 China; 3 School of Computer Science and Engineering, Nanyang Technological University, Singapore, 639798 Singapore

**Keywords:** Disease, miRNA, miRNA-disease association prediction, Feature selection, Random forest

## Abstract

**Background:**

A large body of evidence shows that miRNA regulates the expression of its target genes at post-transcriptional level and the dysregulation of miRNA is related to many complex human diseases. Accurately discovering disease-related miRNAs is conductive to the exploring of the pathogenesis and treatment of diseases. However, because of the limitation of time-consuming and expensive experimental methods, predicting miRNA-disease associations by computational models has become a more economical and effective mean.

**Results:**

Inspired by the work of predecessors, we proposed an improved computational model based on random forest (RF) for identifying miRNA-disease associations (IRFMDA). First, the integrated similarity of diseases and the integrated similarity of miRNAs were calculated by combining the semantic similarity and Gaussian interaction profile kernel (GIPK) similarity of diseases, the functional similarity and GIPK similarity of miRNAs, respectively. Then, the integrated similarity of diseases and the integrated similarity of miRNAs were combined to represent each miRNA-disease relationship pair. Next, the miRNA-disease relationship pairs contained in the HMDD (v2.0) database were considered positive samples, and the randomly constructed miRNA-disease relationship pairs not included in HMDD (v2.0) were considered negative samples. Next, the feature selection based on the variable importance score of RF was performed to choose more useful features to represent samples to optimize the model’s ability of inferring miRNA-disease associations. Finally, a RF regression model was trained on reduced sample space to score the unknown miRNA-disease associations. The AUCs of IRFMDA under local leave-one-out cross-validation (LOOCV), global LOOCV and 5-fold cross-validation achieved 0.8728, 0.9398 and 0.9363, which were better than several excellent models for predicting miRNA-disease associations. Moreover, case studies on oesophageal cancer, lymphoma and lung cancer showed that 94 (oesophageal cancer), 98 (lymphoma) and 100 (lung cancer) of the top 100 disease-associated miRNAs predicted by IRFMDA were supported by the experimental data in the dbDEMC (v2.0) database.

**Conclusions:**

Cross-validation and case studies demonstrated that IRFMDA is an excellent miRNA-disease association prediction model, and can provide guidance and help for experimental studies on the regulatory mechanism of miRNAs in complex human diseases in the future.

## Background

As a category of short non-coding RNA molecules (approximately 22 nt in size), microRNAs (miRNAs) perform regulatory functions by inhibiting target genes translation or directly degrading target genes [1–3]. Since the first miRNA named lin-4 [4] was identified in the 1990s, accumulated evidence has indicated that miRNAs play important molecular functions as gene regulators in various key life activities, including cell differentiation, proliferation and apoptosis, and immune response [5–8]. Furthermore, increasing evidence demonstrated that the abnormal regulation of miRNAs caused the occurrence and progress of many complex human diseases, including various cancers [9–12], cardiovascular diseases [13–15], and metabolic diseases [16–18], just to name a few. To date, tens of thousands of associations between diseases and miRNAs have been discovered and validated by various biological experiments. For example, the human microRNA disease database (HMDD) (v3.2) collected 35,547 experiment-supported associations between 893 diseases and 1206 miRNAs from 19,280 papers [19]. However, the mechanism of miRNA regulation in many complex diseases is still unclear. Therefore, it is very important to discovery and validate more miRNA-disease associations for exploring the pathogenesis and treatment options of these diseases.

To overcome the limitation of high-cost and time-consuming biological experimental methods, researchers have developed many excellent miRNA-disease association computational models in the past decade [20]. The typical miRNA-disease association prediction methods are score function-based models, which prioritize potential miRNA-disease associations using score function by calculating the statistical or distribution characteristics of disease- and miRNA-related information [20]. Based on the supposition that miRNAs with analogous function are inclined to be related to diseases with analogous phenotype, Jiang et al. developed the first computational model for predicting miRNA-disease associations, which combined the functional similarity network of miRNAs and the experiment-validated associations between miRNAs and diseases [21]. However, because of the high false positive rate and false negative rate of the software for predicting miRNA’s target genes [22, 23], the prediction performance of this model is limited. By combining the associations between miRNAs and proteins and the associations between proteins and diseases, Mørk et al. developed a computational model (miRPD), which used the predicted miRNA-target relationships to identify the miRNA-disease associations with medium-confidence, and used the experiment-supported miRNA-target relationships to identify the miRNA-disease associations with high-confidence, respectively [24]. Under the assumption that phenotype-related diseases have similar molecular mechanisms, Xu et al. proposed a computational model for predicting associations between miRNAs and diseases by integrating the experiment-supported associations between diseases and genes and the inferred interactions between miRNAs and target genes [25]. By combining the functional similarity and GIPK similarity of miRNAs, and the semantic similarity and GIPK similarity of diseases, Chen et al. implemented a model named WBSMDA for predicting miRNA-disease associations by computing *within scores* of the experiment-validated miRNA-disease associations and *between scores* of the unverified miRNA-disease associations, which could predict not only diseases associated with new miRNAs but also miRNAs associated with new diseases [26].

Another type of popular methods for predicting miRNA-disease associations are complex network algorithm-based models [20]. Chen et al. predicted disease-associated miRNAs by implementing a random walk with restart (RWRMDA) on the functional similarity network of miRNAs, which used the known disease-associated miRNAs as seed miRNAs, and used a random walk with restart to search potential disease-associated miRNAs [27]. RWRMDA cannot be used to novel diseases which have not experiment-supported associated miRNAs [20]. Xuan et al. also developed a random walk-based mode for miRNA-disease association prediction (MIDP). For diseases with some known associated miRNAs, MIDP predicted potential disease-associated miRNAs by integrating various ranges of topologies around labelled nodes and unlabelled nodes with different transitions; for disease without any known associated miRNAs, MIDP predicted potential miRNAs associated with diseases by integrating the semantic similarity of diseases, the functional similarity of miRNAs, the topological characteristics of miRNA-disease network and the experiment-supported miRNA-disease associations [28]. In addition, Chen et al. constructed a model based on heterogeneous graph inference for predicting miRNA-disease associations (HGIMDA) by combining the functional similarity of miRNAs, the semantic similarity of diseases, the GIPK similarity of miRNAs and diseases, and the experiment-supported miRNA-disease associations [29]. Both MIDP and HGIMDA apply to new diseases which have not experiment-supported associated miRNAs. Recently, Zeng et al. implemented a structural perturbation-based model (SPM) for predicting miRNA-disease associations, which integrated the disease similarity, the miRNA similarity and the associations between miRNAs and diseases into a bilayer network, and measured the link predictability of the network by structural consistency [30]. In addition, Chen et al. constructed a model based on bipartite network projection for predicting miRNA-disease associations (BNPMDA) by combining the integrated similarity of diseases, the integrated similarity of miRNAs and the experiment-supported associations between miRNAs and diseases [31]. Moreover, Chen et al. implemented a model based on matrix decomposition and heterogeneous graph for predicting miRNA-disease associations (MDHGI) by combining the semantic similarity and GIPK similarity of diseases, the functional similarity and GIPK similarity of miRNAs, and the association probability predicted by the sparse learning-based matrix decomposition [32]. MDHGI improved the prediction performance by make the best use of matrix decomposition before heterogeneous network building.

The machine learning-based models are the third most commonly used miRNA-disease association prediction methods [20]. Under the supposition that miRNAs with analogous function is inclined to be associated with diseases with analogous phenotype and vice versa [33], Xuan et al. developed a model based on weighted *k* most similar neighbour for predicting miRNA-disease associations (HDMP), which measured the functional similarity of miRNAs by integrating the phenotype similarity of diseases and the disease terms contents [34]. HDMP improved the prediction performance by integrating the cluster or family information of miRNAs, but it is invalid for diseases which have not experiment-supported associated miRNAs [20]. To overcome the dependence of supervised learning methods on negative sample, Chen et al. constructed a model based on regularized least squares for predicting miRNA-disease associations (RLSMDA), which could be used for predicting new diseases associated miRNAs without negative samples [35]. Later, Chen et al. implemented a model based on restricted Boltzmann machine for predicting both miRNA-disease associations and types of association (RBMMMDA) [36]. Thereafter, Pasquier et al. constructed a computational model named MiRAI based on singular value decomposition-based vector space for predicting miRNA-diseases associations, which used a high-dimensional vector space to represent the distributional characteristics of diseases and miRNAs, and used vector similarity to measure associations between miRNAs and diseases [37]. Furthermore, Chen et al. constructed a computational model named RKNNMDA by combining k-nearest-neighbours (KNN) and support vector machine (SVM) ranking model [38]. Moreover, Chen et al. implemented a predicting model for miRNA-disease associations (LRSSLMDA) based on Laplacian regularized sparse subspace learning. First, the graph theoretical features and statistical features of the diseases and miRNAs were projected to a communal subspace; then, a Laplacian regularization was used to maintain the topical structures of the training samples; finally, an L_1_-norm constraint was utilized to choose useful features of the diseases and miRNAs for prediction [39]. In addition, Lan et al. implemented a prediction model named KBMF-MDI based on kernelized Bayesian matrix factorization with multiple-kernel learning, which measured similarity of miRNAs by the sequence and function characteristics of miRNAs and measured similarity of diseases by the semantic and functional characteristics of disease [40]. Li et al. constructed a predicting model named LPLNS based on label propagation algorithm with linear neighbourhood similarity [41]. Chen et al. constructed a prediction model named IMCMDA based on inductive matrix completion, which completed the missing associations between diseases and miRNAs by the integrated similarity of diseases, the integrated similarity of miRNAs and the experiment-supported associations between diseases and miRNAs [42].

Recently, several excellent machine learning-based prediction models for miRNA-disease associations have been implemented. Zhao et al. developed an adaptive boosting-based model (ABMDA), which utilized k-means clustering-based random sampling on negative samples to balance the positive and negative samples, and used the weight-based weak classifier integration to improve the accuracy of a certain machine learning algorithm [43]. Niu et al. constructed a prediction model based on random walk and binary regression (RWBRMDA), which extracted the features of miRNAs by a random walk with restart, and applied a binary logistic regression to score novel miRNA-disease associations [44]. Peng et al. implemented a convolutional neural network-based framework named MDA-CNN for predicting associations between miRNAs and diseases by combining the similarity between miRNAs, the similarity between diseases and the interactions between proteins [45]. By combining the topological characteristics, the statistical information, and the matrix factorization results for miRNAs and diseases, Chen et al. constructed a decision tree ensemble-based model (EDTMDA), which used randomly selected features and negative samples to trained multiple decision trees, and scored miRNA-disease associations using an average strategy of these decision trees [46]. Moreover, Chen et al. constructed a RF-based model for predicting miRNA-disease associations (RFMDA). First, RFMDA integrated the semantic similarity of diseases, the functional similarity of miRNAs and the GIPK similarity of diseases and miRNAs to represent training samples; then, it implemented feature selection based on the frequency of features in positive and negative samples to lower the dimensionality of the sample space; finally, it trained a RF model to score associations between diseases and miRNAs [47].

Inspired by Chen et al.’s work [47], we proposed an improved RF-based model named IRFMDA for predicting associations between miRNAs and miRNAs. In contrast to the RFMDA of Chen et al., IRFMDA implemented feature selection using the RF variable importance score. Because the variable importance score of RF considers not only the effect of an individual feature on the sample prediction but also the joint effect of multiple features on sample prediction, IRFMDA can more effectively reduce the influence of redundant and noise information to select more meaningful features to represent samples, and this can improve the prediction ability of the model. The experimental results showed that the AUCs of IRFMDA achieved 0.8728, 0.9398 and 0.9363 under local LOOCV, global LOOCV and 5-fold cross-validation, respectively, which over-performed RFMDA and several other excellent prediction models. Case studies on oesophageal cancer, lymphoma and lung cancer showed that 94 (oesophageal cancer), 98 (lymphoma) and 98 (lung cancer) of the top 100 disease-associated miRNAs predicted by IRFMDA were supported by the records in the dbDEMC (v2.0) database. The evaluation results indicated that IRFMDA was an excellent miRNA-disease association prediction model.

## Results

### Feature selection results

**Fig. 1:**
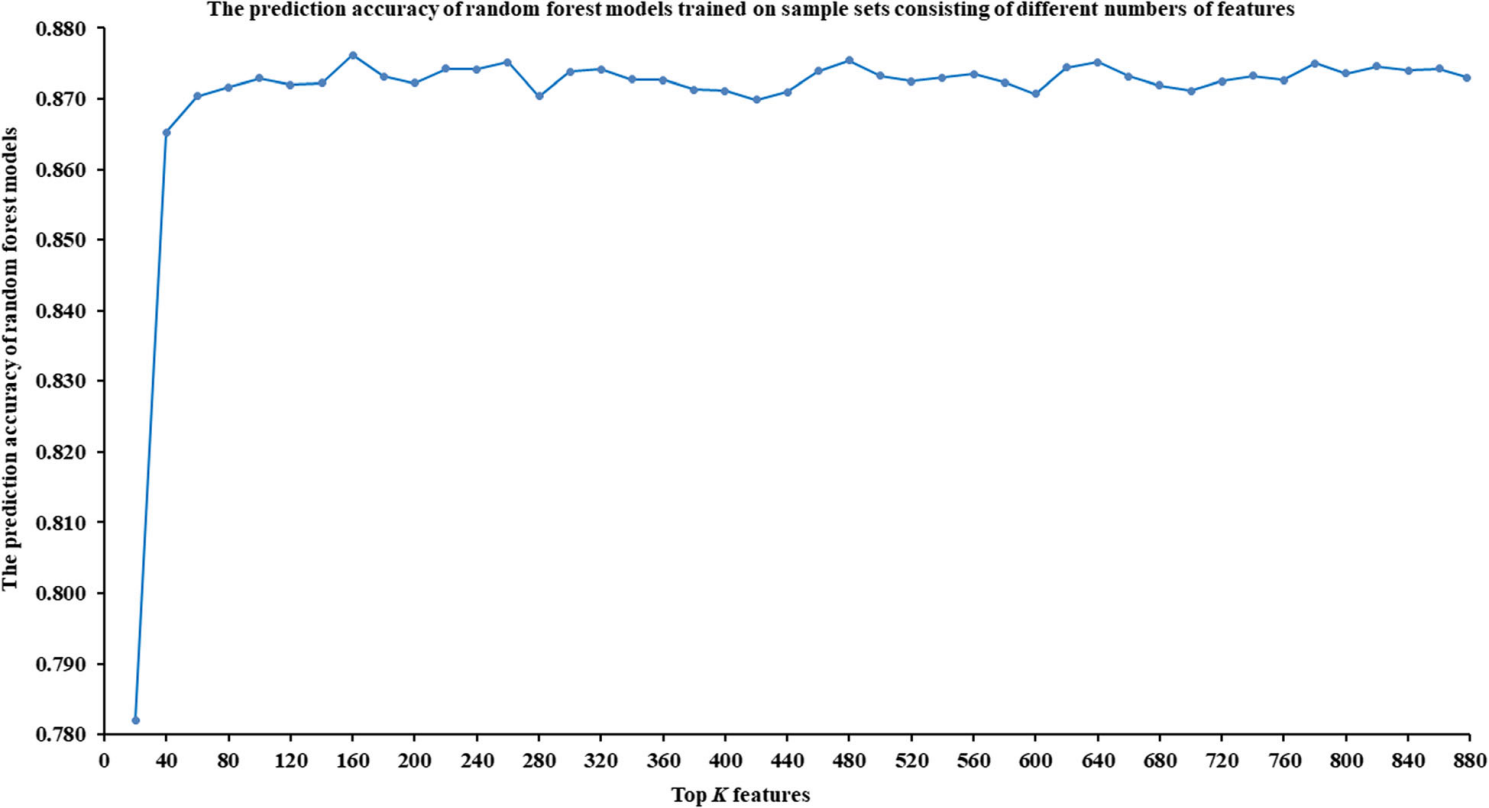
The prediction accuracy of RF models trained on sample sets consisting of different numbers of features. As one can see, the prediction accuracy of the RF model gradually stabilized after the top 100 features were included, and achieved a maximum of 0.876 on the training sample set consisting of the top 160 features

**Fig. 2:**
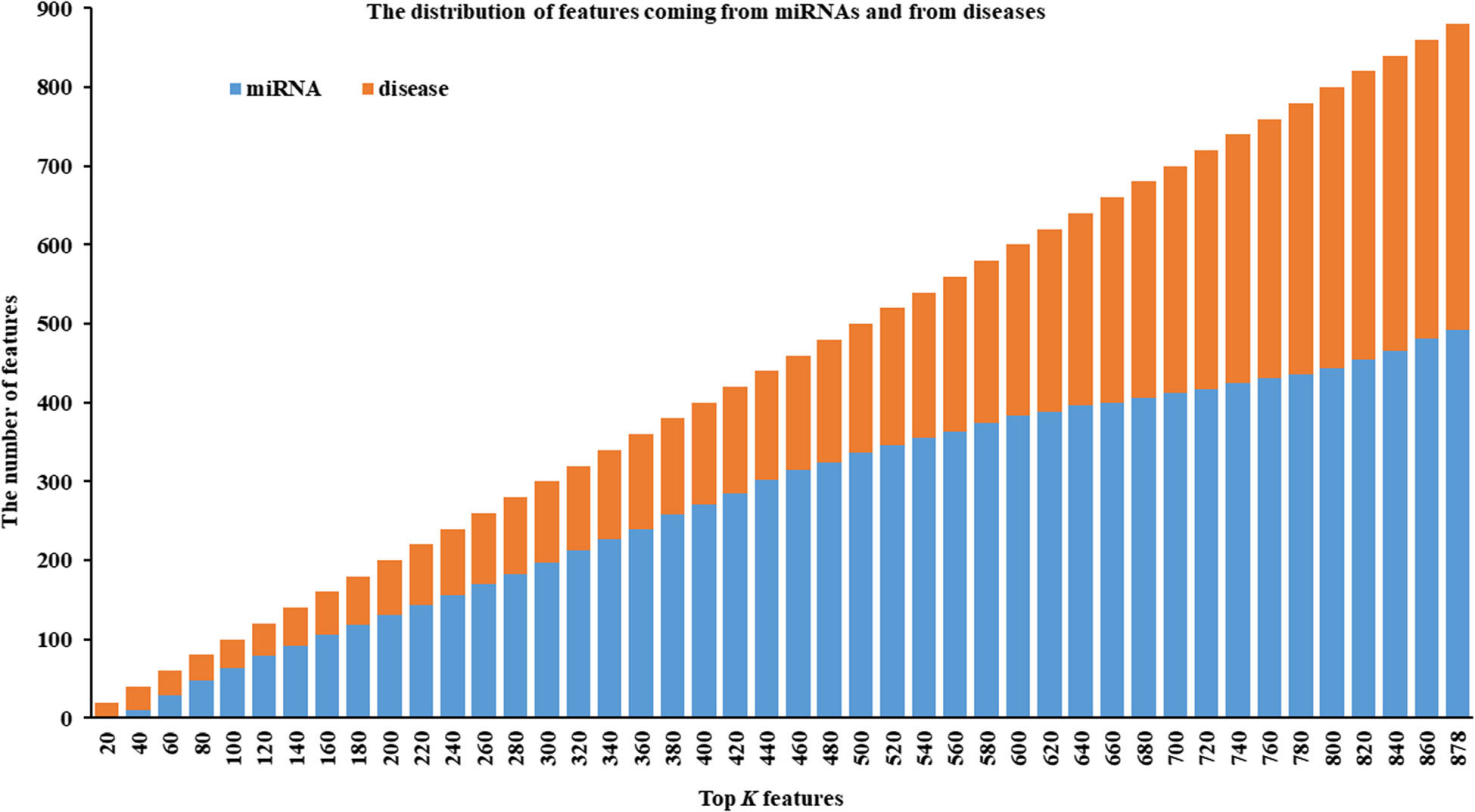
The distribution of features coming from miRNAs and from diseases. As one can see, the distribution of the features coming from miRNAs and from diseases in the top *K* features is uniform, considering the number of miRNA and disease

Feature selection can reduce the computational cost while improve the prediction ability of the machine learning algorithm. In this work, we have explored how many and which features should be used for training prediction models through experiments. We ranked all features in descending order according to their variable importance scores of RF (see Additional file 1: Table S1); and selected the top 20, top 40, …, top 860 and all 878 features to train RF models. To ensure the reliability of the results, we used 10-fold cross-validation to train and test the model, and the average prediction accuracy of the prediction models was computed. The average prediction accuracy of RF models that were trained on sample sets consisting of the top 20, top 40, …, top 860 and all 878 features are shown in Fig. 1 and Additional file 2: Table S2. In addition, to further explore the rationality of the feature selection based on the variable importance score of RF, we counted the distribution of features coming from miRNA and disease in the top 20, top 40, …, top 860 and all 878 features, as shown in Fig. 2.

As shown in Fig. 1 and Additional file 2: Table S2, the prediction accuracy of the RF model gradually stabilized after the top 100 features were included, and achieved a maximum of 0.876 on the training sample set consisting of the top 160 features. Considering the prediction accuracy and training time of the model, we chose the sample set consisting of the top 100 features (see Additional file 3: Table S3) to train the IRFMDA model in this work. Moreover, from Fig. 2 and Additional file 1: Table S1, we can see that the distribution of the features coming from miRNAs and from diseases in the top *K* features is uniform, considering the number of miRNA and disease. This indicates that it is reasonable to select suitable features to represent samples based on variable importance scores.

### Performance evaluation

Referring to the literature 47, we appraise the prediction ability of IRFMDA by local LOOCV, global LOOCV and 5-fold cross-validation. All cross-validations were implemented by utilizing the 5430 experiment-supported associations between 383 diseases and 495 miRNAs in the HMDD (v2.0) database. In global LOOCV and local LOOCV, the positive samples are the 5430 experiment-supported miRNA-disease associations, while all unconfirmed miRNA-disease relationship pairs were taken as unlabelled samples. In each cross-validation, each positive sample was alternately used as a test sample, and the remaining positive samples were used to construct IRFMDA model, which was further used to score the test sample and all unlabelled samples. For global LOOCV, all unlabelled samples and the test sample were sorted together in descending order according to their scores, and then the ranking of the test sample was determined. For local LOOCV, only the unlabelled samples with the same disease as the test sample were sorted together with the test sample according to their scores. For 5-fold cross-validation, the 5430 positive samples were evenly divided into 5 parts, and each part was alternately used as test samples while the remaining four parts were used to train the prediction model. Each test sample in each cross-validation was sorted with all unlabelled samples by their scores. No matter what kind of cross-validation, 5430 rankings of the test samples were obtained eventually in this way. In particular, we repeated 100 runs to ensure the reliability of the results in 5-fold cross-validation.

Like most studies, we evaluated the prediction ability of different prediction models by the area under the receiver operating characteristics (ROC) curve (AUC). The larger the AUC, the better the model. In this work, we used all unlabelled samples as negative samples, and each of them was given a predicted score by IRFMDA. Then, all negative samples were sorted by their predicted scores and combined with positive samples to compute the true positive rate (*TPR*) and the false positive rate (*FPR*) with different thresholds. *TPR* represents the ratio of the actual positive samples in the predicted positive samples (the test samples that were ranked ahead of the specific threshold) to all positive samples, while *FPR* represents the ratio of the actual negative samples in the predicted positive samples (the negative samples that were ranked ahead of the specific threshold) to all negative samples. *TPR* and *FPR* can be calculated by eqs. 1 and 2, respectively.

1 $$ TPR=\frac{TP}{TP+ FN} $$

2 $$ FPR=\frac{FP}{FP+ TN} $$

where *TP* (true positive) indicates that a sample is positive and is predicted to be positive; *FN* (false negative) indicates that a sample is positive and is predicted to be negative; *FP* (false positive) indicates that a sample is negative and is predicted to be positive; *TN* (true negative) indicates that a sample is negative and is predicted to be negative. Finally, the AUC can be computed according to the *TPR* and the *FPR* with different thresholds.

The experimental results of different miRNA-disease association prediction models are shown in Table 1. The AUCs of IRFMDA under local LOOCV, global LOOCV and 5-fold cross-validation achieved 0.8728, 0.9398 and 0.9363 respectively, that were obviously higher than all the models participating in the comparison. Furthermore, to validate the availability of the feature selection method we proposed, we evaluated the prediction performance of RF on sample set consisting of all 878 features. As a result, RF implemented an AUC of 0.7713 under 5-fold cross validation, that is significantly lower than IRFMDA. The experimental results indicated that feature selection based on variable importance score of RF can effectively improve the prediction performance of RF. The comparison results showed that IRFMDA had excellent ability of miRNA-disease association prediction. As a note, the AUC values of the top 10 prediction models for miRNA-disease associations in Table 1 were derived from reference [47], the AUC values of KBMF-MDI and LPLNS were derived from references [40] and [41], respectively, and “-” represents the AUCs were not provided in the original literature. Specifically, our model and reference [47] utilized 5430 experiment-supported associations between 383 diseases and 495 miRNAs in the HMDD; reference [40] utilized 6084 experiment-supported associations between 329 diseases and 550 miRNAs in the HMDD; reference [41] utilized 4791 experiment-supported associations between 327 diseases and 353 miRNAs in the HMDD. Moreover, 10-fold cross-validation was used in reference [41].

**Table 1:** Comparison of AUC values of different miRNA-disease association prediction models

No.	Algorithm	AUC value		
		Global LOOCV	Local LOOCV	5-fold CV
1	HGIMDA	0.8781	0.8077	–
2	MCMDA	0.8749	0.7718	0.8767
3	MaxFlow	0.8624	0.7774	0.8579
4	RLSMDA	0.8426	0.6953	0.8569
5	HDMP	0.8366	0.7702	0.8432
6	WBSMDA	0.8030	0.8031	0.8185
7	MIDP	–	0.8196	–
8	MiRAI	–	0.6299	–
9	RWRMDA	–	0.7891	–
10	RFMDA	0.8891	0.8323	0.8818
11	KBMF-MDI	–	–	0.8815
12	LPLNS	–	–	0.9127
13	IRFMDA-878	–	–	0.7713
14	IRFMDA-100	0.9398	0.8728	0.9363

### Case studies

To further verify the ability of IRFMDA to predict potential miRNAs associated with diseases, we performed two types of case studies on three cancers. First, we performed case study on oesophageal cancer and lymphoma. Here, the 5430 experiment-supported miRNA-disease associations in the HMDD (v2.0) [48] database were taken as positive samples to train the IRFMDA model. The top 100 of disease-related miRNAs predicted by IRFMDA were validated by the dbDEMC (v2.0) database [49], which stored 2224 abnormal expressed miRNAs in 36 kinds of human cancers identified by high-throughput methods.

Oesophageal cancer and lymphoma are two common types of human cancers. It is well known that early diagnosis and treatment of cancer can extend the survival time of cancer patients. A large number of evidences have demonstrated that the dysregulation of some miRNAs has a critical role in the development of cancer. Here, IRFMDA was used to predict potential miRNAs associated with oesophageal cancer and lymphoma. For oesophageal cancer, 30, 47, 76 and 94 of the top 30, 50, 80 and 100 miRNAs predicted by IRFMDA, were validated by records in the dbDEMC (v2.0) (see Table 2), respectively. For lymphoma, 30, 50, 79 and 98 of the top 30, 50, 80 and 100 miRNAs predicted by IRFMDA, were validated by records in the dbDEMC (v2.0) (see Table 3), respectively. These results indicated that the IRFMDA had a good ability to predict miRNA-disease associations.

**Table 2:** Top 100 esophageal cancer-associated miRNAs predicted by IRFMDA using the experiment-supported miRNA-disease associations in the HMDD (v2.0). The top 1–25, top 26–50, top 51–75, and top 76–100 miRNAs associated with esophageal cancer are listed in the first, third, fifth and seventh column, respectively. As one can see, 30, 47, 76 and 94 of the top 30, top 50, top 80 and top 100 were validated by dbDEMC2.0 database

miRNA	Evidence	miRNA	Evidence	miRNA	Evidence	miRNA	Evidence
hsa-mir-29b	dbDEMC2.0	hsa-mir-224	dbDEMC2.0	hsa-mir-128	dbDEMC2.0	hsa-mir-542	dbDEMC2.0
hsa-mir-17	dbDEMC2.0	hsa-mir-107	dbDEMC2.0	hsa-mir-497	dbDEMC2.0	hsa-mir-122	dbDEMC2.0
hsa-mir-195	dbDEMC2.0	hsa-mir-222	dbDEMC2.0	hsa-let-7e	dbDEMC2.0	hsa-mir-132	dbDEMC2.0
hsa-mir-200b	dbDEMC2.0	hsa-mir-29a	dbDEMC2.0	hsa-mir-302b	unconfirmed	hsa-mir-127	dbDEMC2.0
hsa-mir-125b	dbDEMC2.0	hsa-mir-1	dbDEMC2.0	hsa-mir-378a	dbDEMC2.0	hsa-mir-211	dbDEMC2.0
hsa-mir-146b	dbDEMC2.0	hsa-mir-429	dbDEMC2.0	hsa-mir-204	dbDEMC2.0	hsa-mir-367	dbDEMC2.0
hsa-mir-18a	dbDEMC2.0	hsa-mir-24	dbDEMC2.0	hsa-mir-149	dbDEMC2.0	hsa-mir-371a	dbDEMC2.0
hsa-mir-19b	dbDEMC2.0	hsa-mir-9	dbDEMC2.0	hsa-mir-27b	dbDEMC2.0	hsa-mir-96	dbDEMC2.0
hsa-mir-30a	dbDEMC2.0	hsa-mir-212	unconfirmed	hsa-mir-135a	dbDEMC2.0	hsa-mir-424	dbDEMC2.0
hsa-let-7f	dbDEMC2.0	hsa-mir-106b	dbDEMC2.0	hsa-mir-138	dbDEMC2.0	hsa-mir-191	dbDEMC2.0
hsa-mir-142	dbDEMC2.0	hsa-mir-133b	dbDEMC2.0	hsa-mir-372	dbDEMC2.0	hsa-mir-449a	dbDEMC2.0
hsa-mir-181a	dbDEMC2.0	hsa-mir-10b	dbDEMC2.0	hsa-mir-504	dbDEMC2.0	hsa-mir-32	dbDEMC2.0
hsa-mir-218	dbDEMC2.0	hsa-mir-30c	dbDEMC2.0	hsa-mir-328	dbDEMC2.0	hsa-mir-185	dbDEMC2.0
hsa-mir-199b	dbDEMC2.0	hsa-mir-181b	dbDEMC2.0	hsa-mir-30e	dbDEMC2.0	hsa-mir-95	dbDEMC2.0
hsa-mir-16	dbDEMC2.0	hsa-mir-15b	dbDEMC2.0	hsa-mir-23b	dbDEMC2.0	hsa-mir-302e	unconfirmed
hsa-mir-106a	dbDEMC2.0	hsa-mir-125a	dbDEMC2.0	hsa-mir-152	dbDEMC2.0	hsa-mir-323a	dbDEMC2.0
hsa-mir-221	dbDEMC2.0	hsa-mir-206	dbDEMC2.0	hsa-mir-92b	dbDEMC2.0	hsa-mir-483	dbDEMC2.0
hsa-mir-93	dbDEMC2.0	hsa-mir-20b	dbDEMC2.0	hsa-mir-184	dbDEMC2.0	hsa-mir-519a	dbDEMC2.0
hsa-mir-18b	dbDEMC2.0	hsa-mir-373	dbDEMC2.0	hsa-mir-302d	dbDEMC2.0	hsa-mir-208a	unconfirmed
hsa-let-7d	dbDEMC2.0	hsa-mir-140	dbDEMC2.0	hsa-mir-885	dbDEMC2.0	hsa-mir-134	dbDEMC2.0
hsa-mir-124	dbDEMC2.0	hsa-mir-137	unconfirmed	hsa-mir-338	dbDEMC2.0	hsa-mir-23a	dbDEMC2.0
hsa-let-7 g	dbDEMC2.0	hsa-mir-10a	dbDEMC2.0	hsa-mir-491	dbDEMC2.0	hsa-mir-489	dbDEMC2.0
hsa-mir-182	dbDEMC2.0	hsa-mir-26b	dbDEMC2.0	hsa-mir-139	dbDEMC2.0	hsa-mir-197	dbDEMC2.0
hsa-mir-7	dbDEMC2.0	hsa-mir-302c	unconfirmed	hsa-mir-151a	dbDEMC2.0	hsa-mir-326	dbDEMC2.0
hsa-let-7i	dbDEMC2.0	hsa-mir-193b	dbDEMC2.0	hsa-mir-181c	dbDEMC2.0	hsa-mir-495	dbDEMC2.0

**Table 3:** Top 100 lymphoma-associated miRNAs predicted by IRFMDA using the experiment-supported miRNA-disease associations in the HMDD (v2.0). The top 1–25, top 26–50, top 51–75, and top 76–100 miRNAs associated with lymphoma are listed in the first, third, fifth and seventh column, respectively. As one can see, 30, 50, 79 and 98 of the top 30, top 50, top 80 and top 100 were validated by dbDEMC2.0 database

miRNA	Evidence	miRNA	Evidence	miRNA	Evidence	miRNA	Evidence
hsa-let-7b	dbDEMC2.0	hsa-mir-27a	dbDEMC2.0	hsa-mir-26b	dbDEMC2.0	hsa-mir-451a	dbDEMC2.0
hsa-mir-199a	dbDEMC2.0	hsa-let-7a	dbDEMC2.0	hsa-mir-107	dbDEMC2.0	hsa-mir-296	dbDEMC2.0
hsa-mir-222	dbDEMC2.0	hsa-mir-133a	dbDEMC2.0	hsa-mir-7	dbDEMC2.0	hsa-mir-302d	dbDEMC2.0
hsa-let-7c	dbDEMC2.0	hsa-mir-106a	dbDEMC2.0	hsa-mir-338	dbDEMC2.0	hsa-mir-137	dbDEMC2.0
hsa-mir-9	dbDEMC2.0	hsa-mir-141	dbDEMC2.0	hsa-mir-193a	dbDEMC2.0	hsa-mir-130b	dbDEMC2.0
hsa-mir-223	dbDEMC2.0	hsa-mir-100	dbDEMC2.0	hsa-mir-29a	dbDEMC2.0	hsa-mir-127	dbDEMC2.0
hsa-mir-143	dbDEMC2.0	hsa-mir-206	dbDEMC2.0	hsa-mir-30a	dbDEMC2.0	hsa-mir-30d	dbDEMC2.0
hsa-mir-183	dbDEMC2.0	hsa-mir-199b	dbDEMC2.0	hsa-mir-25	dbDEMC2.0	hsa-mir-215	dbDEMC2.0
hsa-mir-182	dbDEMC2.0	hsa-mir-192	dbDEMC2.0	hsa-mir-22	dbDEMC2.0	hsa-mir-367	dbDEMC2.0
hsa-mir-34c	dbDEMC2.0	hsa-mir-34b	dbDEMC2.0	hsa-let-7e	dbDEMC2.0	hsa-mir-449a	dbDEMC2.0
hsa-mir-31	dbDEMC2.0	hsa-mir-93	dbDEMC2.0	hsa-mir-148a	dbDEMC2.0	hsa-mir-152	dbDEMC2.0
hsa-mir-375	dbDEMC2.0	hsa-mir-23a	dbDEMC2.0	hsa-mir-194	dbDEMC2.0	hsa-mir-130a	dbDEMC2.0
hsa-let-7i	dbDEMC2.0	hsa-mir-302b	dbDEMC2.0	hsa-mir-302c	dbDEMC2.0	hsa-mir-128	dbDEMC2.0
hsa-mir-146b	dbDEMC2.0	hsa-mir-145	dbDEMC2.0	hsa-mir-193b	dbDEMC2.0	hsa-mir-491	unconfirmed
hsa-mir-205	dbDEMC2.0	hsa-mir-196a	dbDEMC2.0	hsa-mir-302a	dbDEMC2.0	hsa-mir-376a	dbDEMC2.0
hsa-mir-142	dbDEMC2.0	hsa-mir-140	dbDEMC2.0	hsa-mir-30c	dbDEMC2.0	hsa-mir-28	dbDEMC2.0
hsa-let-7 g	dbDEMC2.0	hsa-mir-378a	dbDEMC2.0	hsa-mir-212	dbDEMC2.0	hsa-mir-197	dbDEMC2.0
hsa-let-7f	dbDEMC2.0	hsa-mir-373	dbDEMC2.0	hsa-mir-429	unconfirmed	hsa-mir-99a	dbDEMC2.0
hsa-let-7d	dbDEMC2.0	hsa-mir-34a	dbDEMC2.0	hsa-mir-149	dbDEMC2.0	hsa-mir-320a	dbDEMC2.0
hsa-mir-181b	dbDEMC2.0	hsa-mir-191	dbDEMC2.0	hsa-mir-96	dbDEMC2.0	hsa-mir-23b	dbDEMC2.0
hsa-mir-10b	dbDEMC2.0	hsa-mir-214	dbDEMC2.0	hsa-mir-181c	dbDEMC2.0	hsa-mir-452	dbDEMC2.0
hsa-mir-195	dbDEMC2.0	hsa-mir-196b	dbDEMC2.0	hsa-mir-370	dbDEMC2.0	hsa-mir-663a	dbDEMC2.0
hsa-mir-125b	dbDEMC2.0	hsa-mir-106b	dbDEMC2.0	hsa-mir-204	dbDEMC2.0	hsa-mir-1	dbDEMC2.0
hsa-mir-151a	dbDEMC2.0	hsa-mir-27b	dbDEMC2.0	hsa-mir-29b	dbDEMC2.0	hsa-mir-365a	dbDEMC2.0
hsa-mir-15b	dbDEMC2.0	hsa-mir-30e	dbDEMC2.0	hsa-mir-103a	dbDEMC2.0	hsa-mir-181d	dbDEMC2.0

In additiong to oesophageal cancer and lymphoma, we also used IRFMDA to score miRNAs associated with other 381 diseases in the HMDD (v.2.0), and the full prediction results are presented in Additional file 4: Table S4. The Additional file 4: Table S4 contains three types of contents: names of diseases, names of miRNAs and correlation scores predicted by IRFMDA.

To demonstrate the ability of IRFMDA to predict novel diseases which have not any validated related miRNAs, the second type of case study was performed on lung cancer. First, we trained IRFMDA on a sample set that did not contain any validated associations between miRNA and lung cancer. Then, we scored and sorted all 495 miRNA-lung cancer samples. Finally, we verified the predicted miRNAs associated with lung cancer by the records in the HMDD (v3.0) and the dbDEMC (v2.0) database. As a result, 100 of the predicted top 100 miRNAs associated with lung cancer by IRFMDA were supported by the dbDEMC (v2.0) database, and 80 of the predicted top 100 miRNAs associated with lung cancer by IRFMDA were supported by the HMDD (v3.0) database (see Table 4). The case study on lung cancer fully showed that IRFMDA has an excellent ability to identify miRNAs related to novel diseases.

**Table 4:** Top 100 lung cancer-associated miRNAs predicted by IRFMDA after deleting all validated miRNA-lung cancer associations in the HMDD (v2.0). The top 1–25, top 26–50, top 51–75, and top 76–100 miRNAs associated with lung cancer are listed in the first, third, fifth and seventh column respectively. As one can see, 100 of the top 100 miRNAs associated with lung cancer predicted by IRFMDA were validated by HMDD v3.0 or dbDEMC v2.0. “D” represents “dbDEMC v2.0”, “H” represents “HMDD v3.0”

miRNA	Evidence	miRNA	Evidence	miRNA	Evidence	miRNA	Evidence
hsa-let-7a	D & H	hsa-mir-19b	D & H	hsa-mir-30b	D & H	hsa-mir-449a	D & H
hsa-let-7 g	D & H	hsa-mir-20a	D & H	hsa-let-7b	D & H	hsa-mir-93	D
hsa-mir-124	D & H	hsa-mir-375	D & H	hsa-let-7c	D & H	hsa-let-7f	D & H
hsa-mir-133b	D & H	hsa-mir-486	D & H	hsa-mir-133a	D & H	hsa-mir-107	D & H
hsa-mir-143	D & H	hsa-mir-497	D & H	hsa-mir-142	D & H	hsa-mir-128	D & H
hsa-mir-146b	D & H	hsa-mir-92a	D & H	hsa-mir-200b	D & H	hsa-mir-302c	D
hsa-mir-148a	D & H	hsa-mir-183	D & H	hsa-mir-200a	D & H	hsa-mir-135a	D & H
hsa-mir-181a	D & H	hsa-mir-139	D & H	hsa-mir-146a	D & H	hsa-mir-339	D
hsa-mir-182	D & H	hsa-mir-372	D & H	hsa-mir-221	D & H	hsa-mir-423	D & H
hsa-mir-199a	D & H	hsa-mir-373	D & H	hsa-mir-27a	D & H	hsa-mir-137	D
hsa-mir-223	D & H	hsa-mir-106b	D	hsa-mir-34a	D & H	hsa-mir-520d	D & H
hsa-mir-29c	D & H	hsa-mir-92b	D	hsa-mir-141	D & H	hsa-mir-205	D & H
hsa-mir-31	D & H	hsa-mir-452	D	hsa-mir-16	D & H	hsa-mir-708	D
hsa-mir-34c	D & H	hsa-mir-302d	D	hsa-mir-135b	D & H	hsa-mir-191	D & H
hsa-mir-7	D & H	hsa-let-7d	D & H	hsa-mir-18b	D & H	hsa-mir-378a	D
hsa-mir-15a	D & H	hsa-mir-429	D	hsa-mir-338	D & H	hsa-let-7i	D & H
hsa-mir-195	D & H	hsa-mir-302a	D	hsa-mir-152	D & H	hsa-mir-200c	D & H
hsa-mir-125a	D & H	hsa-mir-32	D & H	hsa-mir-215	D & H	hsa-mir-29b	D & H
hsa-mir-125b	D & H	hsa-mir-1	D & H	hsa-mir-367	D	hsa-mir-224	D & H
hsa-mir-126	D & H	hsa-mir-196a	D & H	hsa-mir-122	D & H	hsa-mir-29a	D & H
hsa-mir-145	D & H	hsa-mir-25	D	hsa-mir-134	D & H	hsa-mir-30c	D & H
hsa-mir-155	D & H	hsa-mir-34b	D	hsa-mir-130a	D & H	hsa-mir-140	D & H
hsa-mir-17	D & H	hsa-mir-342	D	hsa-mir-574	D & H	hsa-mir-193a	D & H
hsa-mir-18a	D & H	hsa-mir-218	D	hsa-mir-206	D & H	hsa-mir-193b	D
hsa-mir-199b	D & H	hsa-mir-328	D	hsa-mir-204	D	hsa-mir-30e	D & H

## Discussion

Since the discovery of the first miRNA, numerous experiments have demonstrated that the abnormal regulation of miRNAs is closely associated with many complex human diseases. MiRNA-disease association identification is key for exploring the pathogenesis and treatment options of diseases. However, it is not only high cost but also time consuming to discover miRNAs associated with diseases by biological experiments. Therefore, researchers developed a number of computational models to predict disease-related miRNAs. Inspired by Chen et al.’s work [47], we developed an IRFMDA model based on RF to predict potential miRNA-disease associations.

Different from RFMDA proposed by Chen et al. [47], IRFMDA implemented a feature selection based on the variable importance score of RF, which can reduce the influence of redundant and noise information on sample prediction and improve the prediction ability of the RF. In terms of AUCs under three types of cross-validation, IRFMDA is significantly better than several excellent models, such as RFMDA, KBMF-MDI and LPLNS. Moreover, case studies on oesophageal cancer, lymphoma and lung cancer further demonstrate that IRFMDA is a better and reliable prediction model.

Through analysis, we identified several factors that enable IRFMDA to achieve excellent performance. First, IRFMDA represents miRNA-disease samples by the feature vector that integrates experiment-supported miRNA-disease associations, the semantic similarity of diseases, the functional similarity of miRNAs and the GIPK similarity of diseases and miRNAs. Second, IRFMDA implements feature selection based on the variable importance score of RF, which considers not only the effect of an individual feature on the sample prediction but also the joint effect of multiple features on sample prediction. Finally, RF can implement an unbiased generalization error estimator which makes IRFMDA achieve good generalization performance.

There are several limitations to IRFMDA. First, IRFMDA is a supervised machine learning model, which requires both positive samples and negative samples. However, negative samples are usually unavailable for predicting miRNA-disease associations. The negative samples constructed by randomly selecting unverified miRNA-disease associations may weaken the prediction ability of IRFMDA. In addition, the limited knowledge of miRNA-disease association may constrain the prediction performance of IRFMDA. Furthermore, except for miRNA and disease similarity, more miRNA- and disease-related information may be integrated to train RF model in next work. Therefore, we will attempt to improve IRFMDA to obtain better prediction performance in the future.

## Conclusions

To identify disease-associated miRNAs is important for exploring the mechanism of miRNAs in diseases. Predicting miRNA-disease associations by computational methods can provide guidance for biological experiments. Inspired by the work of predecessors, we proposed an improved RF-based prediction model for miRNA-disease associations (IRFMDA). First, IRFMDA represented training samples by feature vector integrating the disease semantic similarity, the disease GIPK similarity, the miRNA functional similarity and the miRNA GIPK similarity. Then, IRFMDA implemented feature selection based on variable importance score of RF to choose more useful features to train prediction model. Finally, IRFMDA trained RF regression model to score potential miRNA-disease associations. The AUCs under three kinds of cross-validations, and two kinds of case studies on three cancers, demonstrated that IRFMDA has excellent ability to predict associations between diseases and miRNAs. Therefore, we anticipate that IRFMDA can help researchers perform experimental studies on the regulatory role of miRNAs in complex human diseases.

## Methods

### Experiment-supported miRNA-disease associations

First, we obtained the 5430 experiment-supported miRNA-disease associations from the HMDD (v2.0) database [48], which covered 495 miRNAs and 383 diseases. Then, an *nd* × *nm* adjacency matrix *DMAM* was constructed, where *nd* (=383) represents the number of rows (diseases) and *nm* (=495) represents the number of columns (miRNAs). The value of the element *DMAM*(*d*(*i*), *m*(*j*)) was set as 1 when disease *d*(*i*) was validated to be associated with miRNA *m*(*j*) by experiments; otherwise, 0.

### Functional similarity of miRNAs

Under the supposition that miRNAs with analogous functions are inclined to be related to diseases with analogous phenotypes and vice versa, the functional similarity score between two miRNAs could be computed. First, we obtained the functional similarity of 495 miRNAs from Cui’s lab website [33]. Next, we built a 495 × 495 miRNA similarity matrix *MFSM*, where the value of the element *MFSM*(*m*(*i*), *m*(*j*)) was set as the functional similarity score between *m* (*i*) and *m*(*j*) miRNAs.

### Semantic similarity score 1 of diseases

The semantic similarity of diseases was calculated based on MeSH [50] descriptors by Chen et al.’s method [47]. According to MeSH descriptors, we first constructed a directed acyclic graph (DAG) for a disease *D*. In a DAG(D), the vertexes consist of the disease *D* and its ancestral vertex, and each directed edges indicates a connection from the parent vertex to the child vertex [47]. Based on the DAG(D), the semantic score of a disease *D* is calculated by eq. 3.

3 $$ DS1(D)=\sum \limits_{d\in S(D)} CS{1}_D(d) $$

where *S*(*D*) represents a collection of all vertexes of *DAG(D)*, and *CS*1_*D*_(*d*) represents the score that a disease *d* in *DAG(D)* contributes to the semantic value of the disease *D* and is calculated by eq. 4.

4 $$ \left\{\begin{array}{c} CS{1}_D(d)=1\kern19.5em if\ d=D\\ {} CS{1}_D(d)=\mathit{\max}\left\{\Delta  \ast CS{1}_D\left({d}^{\prime}\right)|{d}^{\prime}\in children\ of\ d\right\}\kern1.25em if\ d\ne D\end{array}\right. $$

Here, *∆* is the semantic contribution attenuation coefficient. As seen from eq. 2, the contribution score of disease *D* to itself is equal to 1, while the contribution score of other diseases to disease *D* decreased as the length between disease *D* and the other diseases increased. In this article, *∆* was set as 0.5 based on previous studies [33].

Based on the supposition that the larger the DAGs area they share, the more similar two diseases, the semantic similarity score 1 between *d*(*i*) and *d*(*j*) is calculated by eq. 5.

5 $$ DSS1\left(d(i),d(j)\ \right)=\frac{\sum_{d\in S\left(\mathrm{d}(i)\ \right)\cap S\left(\mathrm{d}(j)\ \right)}\left( CS{1}_{\mathrm{d}(i)}(d)+ CS{1}_{\mathrm{d}(j)}(d)\right)}{DS1\left(d(i)\right)+ DS1\left(d(j)\right)} $$

According to eq. 5, we constructed a 383 × 383 disease semantic similarity matrix *DSS*1 in which the element *DSS*1(*d*(*i*), *d*(*j*) ) represents the semantic similarity score 1 between *d*(*i*) and *d*(*j*) diseases.

### Semantic similarity score 2 of diseases

For the semantic similarity score 1 of diseases, if two or more diseases are located at the same layer of *DAG(D)*, their contributions to the semantic similarity score of *D* are the same. However, that is not the case. If some diseases exist in different amount of DAGs, then their contributions may be different. In this case, the diseases existing in more DAGs may have less contributions than those existing in fewer DAGs [37]. Therefore, we introduced a second disease semantic similarity model. Adopting Xuan et al.’s method [31], the contribution score of a disease *d* in *DAG(D)* to the semantic value of disease *D* is calculated by eq. 6.

6 $$ CS{2}_D(d)=-\mathit{\log}\left(\frac{the\ number\ of\ DAGs\ including\ d}{the\ number\ of\ diseases}\right) $$

Accordingly, the semantic score of disease D is calculated by eq. 7.

7 $$ DS2(D)=\sum \limits_{d\in S(D)} CS{2}_D(d) $$

where *S*(*D*) represents the vertex set of *DAG(D)*. Then, the semantic similarity score 2 between *d*(*i*) and *d*(*j*) is calculated by eq. 8.

8 $$ DSS2\left(d(i),d(j)\ \right)=\frac{\sum_{d\in S\left(\mathrm{d}(i)\ \right)\cap S\left(\mathrm{d}(j)\ \right)}\left( CS{2}_{\mathrm{d}(i)}(d)+ CS{2}_{\mathrm{d}(j)}(d)\right)}{DS2\left(d(i)\right)+ DS2\left(d(j)\right)} $$

Similarly, according to eq. 8, we constructed a 383 × 383 matrix *DSS*2 to represent the semantic similarity of diseases, and the element *DSS*2(*d*(*i*), *d*(*j*) ) represents the semantic similarity score 2 between *d*(*i*) and *d*(*j*) diseases.

### Gaussian interaction profile kernel similarity of diseases

Under the supposition that diseases with analogous phenotype are inclined to be associated with miRNAs with analogous function and vice versa, the GIPK similarity of diseases can be calculated [51]. First, we constructed a binary vector *IP*(d(*i*)) to record the associations between disease *d*(*i*) and each of the 495 miRNAs. If there is an experiment-supported association between them, the corresponding element value of *IP*(d(*i*)) is set as 1; otherwise, 0. Then, the GIPK similarity between two disease, *d*(*i*) and *d*(*j*), is computed by eq. 9.

9 $$ DK\mathrm{S}\left(d(i),d(j)\right)=\mathit{\exp}\left(-{\alpha}_d{\left\Vert IP\left(d(i)\right)-\right.\left. IP\left(d(j)\right)\right\Vert}^2\right) $$

where *α*_*d*_ is utilized to adjust the bandwidth of kernel, and can be calculated by normalizing the original bandwidth parameter *α*_*d*_^′^ by eq. 10.

10 $$ {\alpha}_d={\alpha_d}^{\prime }/\left(\frac{1}{nd}{\sum}_{i=1}^{nd}{\left\Vert IP\left(\mathrm{d}(i)\right)\right\Vert}^2\right) $$

where *nd* represents the number of all diseases studied. According to the previous study [43], the value of *α*_*d*_^′^ here is 1.

### Gaussian interaction profile kernel similarity of miRNAs

Similarly, the GIPK similarity between two miRNAs, *m*(*i*) and *m*(*j*), is computed by eqs. 11 and 12.

11 $$ MKS\left(m(i),m(j)\right)=\mathit{\exp}\left(-{\alpha}_m{\left\Vert IP\left(m(i)\right)-\right.\left. IP\left(m(j)\right)\right\Vert}^2\right) $$

12 $$ {\alpha}_m={\alpha_m}^{\prime }/\left(\frac{1}{nm}{\sum}_{i=1}^{nm}{\left\Vert IP\left(m(i)\right)\right\Vert}^2\right) $$

where *IP*(*m*(*i*)) is a binary vector that records the associations between miRNA *m*(*i*) and each of the 383 diseases. If there is an experiment-supported association between them, the corresponding element value of *IP*(*m*(*i*)) is set as 1; otherwise, 0. Similar to *α*_*d*_^′^, *α*_*m*_^′^ is set as 1 here according to previous study [43].

### Integrated similarity of diseases

According to Chen et al.’s method [25, 33, 37], we constructed an integrated disease similarity matrix *IDSM* by integrating the semantic similarity score 1, the semantic similarity score 2 and the GIPK similarity of diseases. The element *IDSM*(*d*(*i*), *d*(*j*) ) of the *IDSM* is computed by equation 13, which indicates the integrated disease similarity between *d*(*i*) and *d*(*j*) diseases.

13 $$ IDSM\left(d(i),d(j)\right)=\left\{\begin{array}{c}\frac{DSS1\left(d(i),d(j)\ \right)+ DSS2\left(d(i),d(j)\ \right)}{2}\kern1.25em if\ d(i)\  and\ d(j)\  have\ semantic\ similarity\\ {}\  DKS\left(d(i),d(j)\right)\kern6.5em otherwise\kern16em \end{array}\right. $$

where *d*(*i*) and *d*(*j*) have semantic similarity if both *d*(*i*) and *d*(*j*) have their own DAGs.

### Integrated similarity of miRNAs

Similarly, we integrated the functional similarity and the GIPK similarity of miRNAs to construct an integrated similarity matrix of miRNA, named *IMSM*. The element *IMSM*(*m*(*i*), *m*(*j*) ) of the *IMSM* is computed by equation 14, which represents the integrated similarity between *m*(*i*) and *m*(*j*) miRNAs.

14 $$ IMSM\left(m(i),m(j)\right)=\left\{\begin{array}{c} MFSM\left(m(i),m(j)\right)\kern1.75em if\ m(i)\  and\ m(j)\  have\ functional\ similarity\\ {}\  MKS\left(m(i),m(j)\right)\kern2.5em otherwise\kern17.75em \end{array}\right. $$

### Variable importance score of RF

RF is a popular machine learning algorithm that can be applied for not only classification but also regression [52]. RF integrates bootstrap and random sample splitting techniques. By bootstrap-based random resampling with replacement, many decision trees are trained and integrated into a forest to predict the category or target variable values of unknown samples. In addition, different from the general decision tree model, a given number of input variables are randomly selected to perform a split node at each node, and no pruning step is performed in the process of training decision trees in a RF. Through these techniques, a RF can achieve outstanding and robust performance. Therefore, RF has been widely used in many bioinformatics tasks in the past two decades.

The variable importance score is a characteristic function of RF, which is defined as the average reduction value of classification accuracy before and after minor disturbance of the variable of OOB (out-of-bag) samples. The variable importance score considers not only the individual impact of each variable but also the multivariate interactions with other variables. Given a set of bootstrap sampling *b = 1, 2, …, B*, the importance score *S*_*j*_ of variable *X*_*j*_ can be computed as follows [52]:

1. For *b = 1*, the training sample set is represented by *TSS*_*b*_, and the out-of-bag data are represented by ${L}_b^{oob}$;
2. Train decision tree *T*_*b*_ on *TSS*_*b*_;
3. Use *T*_*b*_ for prediction on ${L}_b^{oob}$, and the prediction accuracy is represented by ${R}_b^{oob}$;
4. Randomly perturb the value of the variable *X*_*j*_ of each sample in ${L}_b^{oob}$ until its association with the target variable is broken, and the perturbed dataset is represented by ${L}_{bj}^{oob}$;
5. Use *T*_*b*_ for prediction on ${L}_{bj}^{oob}$, and the prediction accuracy is represented by ${R}_{bj}^{oob}$; if the original variable is associated with the target variable, the prediction accuracy will reduce substantially.
6. For *b = 2, …, B*, repeat steps 1–5;
7. The importance score *S*_*j*_ of variable *X*_*j*_ is computed by equation 15.

15 $$ {S}_j=\frac{1}{B}{\sum}_1^B\left({R}_b^{oob}-{R}_{bj}^{oob}\right) $$

where the variable *B* represents the times of resampling for constructing RF, which corresponds to the *ntree* parameter of the RF algorithm, that is, the number of decision trees in the forest. *B* or *ntree* should not be set to too small a number, to ensure that every input row is predicted at least a few times. In this work, we use the default value of the randomForest model, that is, *ntree* is set to 500. For more information on the variable importance of RF, see reference [53].

### IRFMDA

**Fig. 3:**
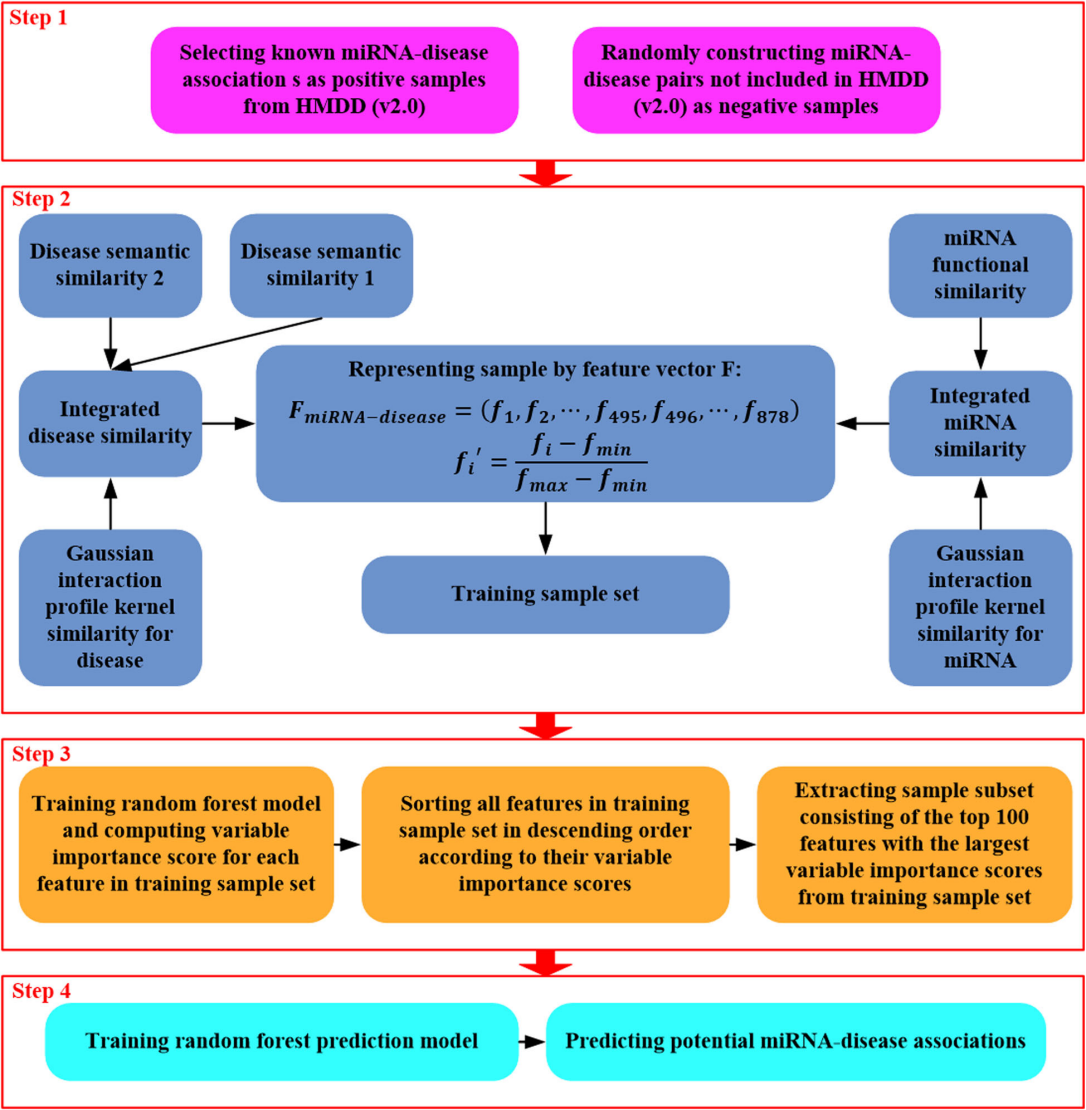
Flowchart of the IRFMDA model for predicting potential associations between miRNAs and diseases

Inspired by RFMDA proposed by Chen et al. [47], in this paper, we implemented an improved RF-based prediction model for miRNA-disease associations (IRFMDA). IRFMDA can be constructed by four steps (see Fig. 3): (1) sample selection; (2) sample representation; (3) feature selection; (4) model training and sample prediction. The biggest difference between RFMDA and IRFMDA lies in the different feature selection method in the third step. Next, we introduce the above steps in detail.

In step 1, we selected the same number of negative samples as the positive samples to construct a training sample set. First, we used 5430 experiment-supported associations between miRNAs and disease from HMDD (v2.0) as positive samples. Then, we randomly selected 5430 pairs of unconfirmed miRNA-disease associations as negative samples. Specifically, 5430 negative samples were selected according to the following method: we first randomly chose a disease from 383 diseases; next, we randomly chose a miRNA from 495 miRNAs; next, we combined the randomly selected disease and miRNA as a negative sample if the combined miRNA-disease association was not contained in HMDD (v2.0); at last, we repeated the above steps until 5430 negative samples were obtained. Finally, we combined the positive samples and the negative samples into a training sample set consisting of 10,860 samples.

In step 2, we constructed a feature vector to represent samples. First, we computed the integrated disease similarity between each pair of diseases and the integrated miRNA similarity between each pair of miRNAs by equations 13 and 14. As a result, we obtained a 383-dimensional vector consisting of 383 integrated disease similarity scores to represent each disease, and a 495-dimensional vector consisting of 495 integrated miRNA similarity scores to represent each miRNA. Then, we represented each sample by an 878-dimensional feature vector consisting of combining the 383 integrated disease similarity score and 495 integrated miRNA similarity score as equation 16.

16 $$ {F}_{miRNA- disease}=\left({f}_1,{f}_2,\cdots, {f}_{495},{f}_{496},\cdots, {f}_{878}\right) $$

where (*f*_1_, *f*_2_, ⋯, *f*_495_) represents the 495 integrated miRNA similarity scores, and (*f*_496_, ⋯, *f*_878_) represents the 383 integrated disease similarity scores. Finally, *f*_*i*_ was normalized to *f*_*i*_^′^ by equation 17.

17 $$ {f_i}^{\prime }=\frac{f_i-{f}_{min}}{f_{max}-{f}_{min}} $$

Where *f*_*min*_ and *f*_*max*_ are the minimum and the maximum of *f*_*i*_ (*i* = 1, 2, …, 878), respectively.

In step 3, we performed feature selection to reduce the interference of the redundant and noise information on sample prediction and to improve the prediction ability of the RF model. Here, we implemented feature selection based on the variable importance score of RF. First, we computed the variable importance score of each feature by training a RF model on a sample set consisting of all 838 features. Then, we ranked all features in descending order according to their variable importance scores. Next, we selected 20, 40, …, 860, 878 features to train the RF model; and finally chose the feature set with the higher prediction accuracy as the final training set. According to the experimental results, we chose the top 100 features with the highest variable importance scores to represent the training samples. To ensure reliability, we adopted 10-fold cross-validation when calculating the variable importance score. The average value of variable importance scores in 10-fold was used to rank the variables. Because the variable importance score of RF considers not only the impact of an individual feature on the response variable but also the interaction of multiple features on the response variable, the feature selection method based on the variable importance score of RF can select more distinguishing features to characterize the sample and improve the prediction performance of the model.

In the last step, we trained a RF prediction model on a training sample set consisting of the top 100 most important features through running the *randomForest* package on the *R* platform. In the training sample set, each sample was recorded as a 100-dimensional vector according to steps 2 and 3, and each positive sample was labelled as 1 while each negative sample was labelled as 0. As a result, we got a RF regression model that could give a score for each unknown miRNA-disease pairs. The larger the score of a miRNA-disease pair, the greater the likelihood of association between the disease and the miRNA. Finally, it is worth noting that the *mtry* and the *ntree*, two parameters of *randomForest*, were set to 33 (the number of features / 3) and 500, respectively, according to the recommended values.

## Supplementary information

**Additional file 1: Table S1.** Variable importance score of 878 features.

**Additional file 2: Table S2.** Prediction accuracy of RF models trained on sample sets consisting of different numbers of features.

**Additional file 3: Table S3.** Training sample set consisting of 100 features.

**Additional file 4: Table S4.** Prediction result for all diseases in HMDD.

## Data Availability

The functional similarity score of miRNAs was downloaded from http://www.cuilab.cn/. The semantic similarity score of diseases was obtained from https://github.com/IMCMDAsourcecode/IMCMDA. The experiment-supported miRNA-disease associations were obtained from HMDD (v2.0) database. The other data used in this article were contained in the article and its additional files.

## Abbreviations

AUC: The area under the ROC curve
dbDEMC: Database of differentially expressed miRNAs in human cancers
FN: False negative
FP: False positive
FPR: False positive rate
HMDD: Human micro disease database
LOOCV: Leave-one-out cross validation
OOB: Outside of bag
RF: Random forest
ROC: Receiver operating characteristics
TN: True negative
TP: True positive
TPR: True positive rate
